# A critical analysis of the impact of endocrine disruptors as a possible etiology of primary ovarian insufficiency

**DOI:** 10.5935/1518-0557.20200005

**Published:** 2020

**Authors:** Cecilia de Souza Monteiro, Erica Becker de Sousa Xavier, João Pedro Junqueira Caetano, Ricardo Mello Marinho

**Affiliations:** 1 Laboratory of Human Reproduction Professor Aroldo Fernando Camargos, Hospital das Clínicas, Federal University of Minas Gerais, Belo Horizonte, MG, Brazil; 2 Pro Criar, Medicina Reprodutiva, Belo Horizonte, MG, Brazil; 3 Faculdade de Ciências Médicas de Minas Gerais, Belo Horizonte, MG, Brazil

**Keywords:** Primary ovarian insufficiency, Occupational exposure, Follicular atresia, Endocrine disruptor

## Abstract

Primary ovarian insufficiency is a cause of infertility that affects about 1% of women under 40 years old, and is considered as idiopathic in 75% of cases. This review aims to carry out a critical synthesis of the knowledge of the chemical agents likely to affect follicular stock in humans and / or animals, by direct toxicity to follicles, or by increasing their recruitments. For the majority of toxic agents, only experimental data are currently available. We propose a strategy to encourage progress in identifying occupational factors responsible for premature ovarian failure.

## INTRODUCTION

### A definition of endocrine disruptors

Women may be exposed to gonadal toxicity when they are in contact with endocrine disrupting chemicals present in food, water, air, or products they use at home or at work. Reproductive safety may be compromised by contaminants present in the environment, including synthetic chemicals and metals that affect reproductive health and development (Strauss *et al*., 2018). Some chemicals are known to affect hormones critical for female endocrine and reproductive development (Wang *et al*., 2016). Compounds known as endocrine disruptors can mimic or antagonize the activity and change the signaling of steroid hormones, thus contributing to adverse ovarian function outcomes (Wang *et al*., 2016). Adequate ovarian cycle function relies on multiple factors, including gonadal exposure to potentially toxic environmental products. Endocrine disruptors may accelerate the genetically predetermined rate of oocyte loss, thereby reducing follicular reserve.

This paper presents the current knowledge on the correlation between exposure to toxic agents and ovarian reserve involvement leading to primary ovarian insufficiency and discusses recommendations to prevent exposure to endocrine disruptors and safeguard gonadal function.

### Magnitude of the problem

Our water, air, soil, and food have been consistently contaminated with chemicals and heavy metals. An explosive increase was seen in the manufacturing and processing of chemicals after World War II, which have amounted to approximately 90,000 chemicals currently in use globally. This is a relevant issue for reproductive health and human health more broadly, as well as for wildlife and the ecosystem (Niederberger *et al.,* 2018).

### Environmental toxicants and reproductive processes

The last two decades saw the publication of numerous research reports and reviews on the impact of endocrine disrupters and other environmental toxicants on animal and human development and reproduction. The data support causal and associative effects of endocrine disrupters on the gonads, the hypothalamic-pituitary gonadal axis, and the development and function of the reproductive tract, all of which are tied to fertility. Associated disorders include aneuploidy, primary ovarian insufficiency, polycystic ovarian syndrome, endometriosis, fibroids, miscarriage, endocrine cancers, lactation, and altered pubertal timing (Niederberger *et al.,* 2018).

Much of the animal data provide information on the mode of action of endocrine disruptors, including their effects on steroid hormone signaling pathways, altered gene expression, and epigenetic alterations (Niederberger *et al.,* 2018). Exposure to environmental chemicals and associated adverse health outcomes are unequally distributed among people, communities, and countries. Some of the differences in vulnerability and risk have been correlated with age, sex, genes, underlying health status, and exposure to other environment stressors (Strauss *et al*., 2018).

### Primary ovarian insufficiency: diagnosis and epidemiology

Proper ovarian function is vital for the production of sex steroids, which by their turn are needed in the development of the genital tract, maintenance of adequate bone mineral density, and general feminine health (Tucker *et al.,* 2016). Primary ovarian insufficiency (POI) is a cause of female infertility affecting about 1% of women under the age of 40. Three quarters of the cases are deemed idiopathic (Béranger *et al*., 2012).

The prevalence of POI is even lower in younger patients, affecting one in 1,000 women under the age of 30 and one in 10,000 women under the age of 20 years (Béranger *et al*., 2012). Diagnosis is based on symptoms, more specifically primary or secondary amenorrhea lasting for more than four months combined with serum levels of follicle stimulating hormone (FSH) above 40IU/mL confirmed by after a second test performed within a month, accompanied by decreased estrogen levels (Béranger *et al*., 2012). POI has been associated with greater morbidity, including psychological distress and increased risk of osteoporosis and cardiovascular disease (Béranger *et al*., 2012). Etiology is identified in only 25% of the cases. Most known causes are of genetic origin, and include Turner syndrome and fragile X syndrome. Acquired forms appear more frequently after radiotherapy or chemotherapy (Béranger *et al*., 2012).

## MATERIAL AND METHODS

All chemicals cited in interventional research and reviews that were associated with reduced ovarian function were included in this review. We searched for publications on databases PubMed, Lilacs, and SciELO in August 2019 using the following keywords: “primary ovarian insufficiency”, "ovarian failure", “ovarian reserve”, "environmental exposure", “occupational exposure”, “endocrine disruptors”, “female reproduction”, “reproduction health”, “in vitro fertilization”, and “infertility”.

The search included randomized controlled trials, meta-analyses, systematic reviews, and prospective cohort studies published between 2010 and 2019 written in English. Studies that investigated chemical exposure in animals or male humans only were excluded. The risk of bias in the selected articles is low, since our search included prospective studies, systematic reviews, and meta-analyses.

**Figure 1:**
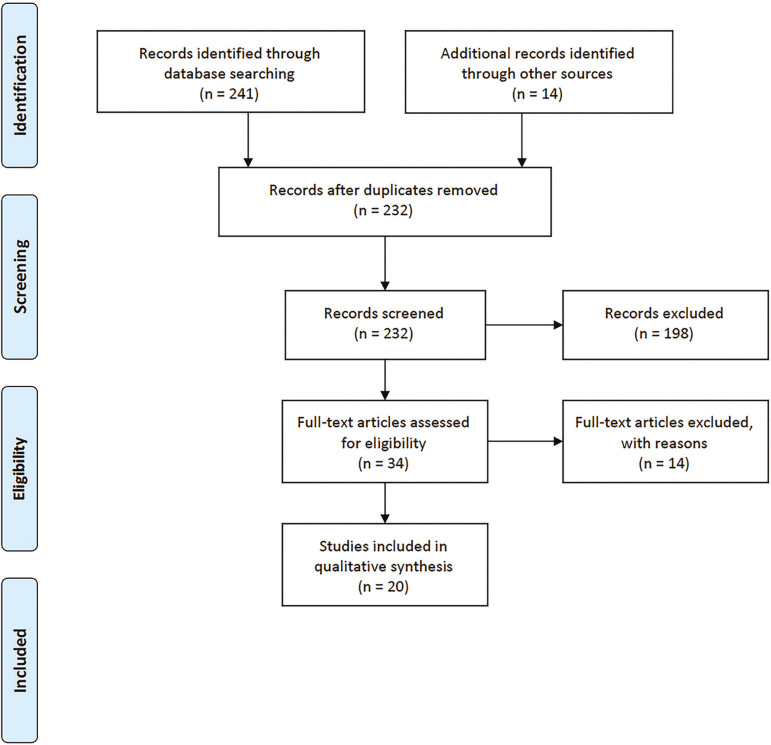
Search Flowchart (Adapted fromMoher *et al*., 2009).

A total of 241 papers were found in the PubMed, Lilacs, and SciELO databases. Another 14 articles from other sources were added. We excluded 235 articles (23 duplicates, 198 records, and 14 full-text articles) and considered 20 papers in our qualitative summary (Figure 1).

**Figure 2:**
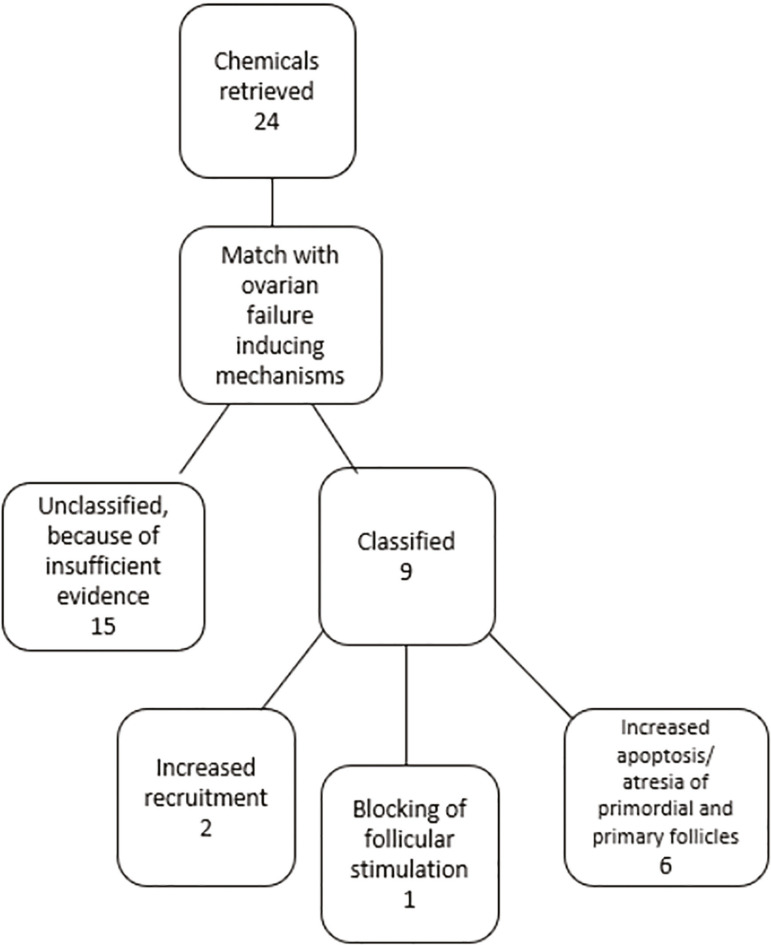
Selection flowchart for papers on the impacts of endocrine disruptors on ovarian physiology (Adapted from Béranger *et al.,* 2012.)

Our search found a total of 24 ovarian endocrine disruptors. Nine of them had well-described modes of action. The described modes of action fell into the following categories: increased recruitment (2); blocking follicle-stimulation hormone (1); and increased apoptosis/atresia of primordial and primary follicles (6) (Figure 2).

## RESULTS

### Most significant studies selected

A prospective study examined the associations between the levels of eight urinary phthalate metabolites in 599 couples submitted to in vitro fertilization (IVF). The results demonstrated that exposure to some phthalates may adversely affect IVF outcomes, particularly when couple exposure was jointly modeled. The associations between IVF outcomes and phthalate metabolites were stronger in women whose phthalate levels were >75^th^ percentile, which may be due to impaired metabolism and excretion (Al-Saleh *et al.*, 2019).

Another prospective study also found an association between urinary concentrations of phthalates and IVF outcomes. The authors evaluated 136 women undergoing IVF in a Tertiary University Affiliated Hospital. Participants provided one to two urine samples per cycle during ovarian stimulation and before oocyte retrieval. The results suggest that phthalates may impair early IVF outcomes and oocyte parameters in particular (Machtinger *et al.*, 2018).

A systematic review and meta-analysis including 97 studies evaluated the association between primary ovarian insufficiency and environmental toxicants. The researchers found that bisphenol A, phthalates, and pesticides most commonly had a negative impact on ovarian function (Niederberger *et al.,* 2018).

A large prospective cohort studies called “The Environment and Reproductive Health (EARTH) Study” investigated the impact of environmental, nutritional, and lifestyle-related parameters of women and men on their fertility and pregnancy outcomes. The study has been in progress since 2004 and has enrolled 799 women seeking fertility evaluation and treatment at a large academic hospital and fertility center. The authors are prospectively collecting a combination of biological samples (blood, urine, semen), self-reported questionnaire data (including a validated food frequency questionnaire), and medical information abstracted from fertility clinic and hospital records. They measured over 40 different biomarkers of exposure to environmental chemicals that may potentially interfere with reproductive health (Hauser *et al.*, 2016; Messerlian *et al.*, 2016a; 2016b; 2018).

A pilot study measured 43 polychlorinated biphenyls (PCBs) in ovarian follicular fluid samples collected from 32 women undergoing in vitro fertilization (IVF). The authors found significant inverse associations between higher levels of PCBs and indicators of ovarian reserve and IVF outcomes (Bloom *et al.*, 2017).

A review evaluated the impact of some endocrine disruptors (phthalates, parabens, triclosan, bisphenol A, organochlorine, and perfluorinated compounds) on female reproductive potential. The study concluded that the endocrine disruptors mentioned above had negative impacts on ovarian reserve markers and in vitro fertilization outcomes (Karwacka *et al.*, 2019).

Another prospective cohort study looked into urinary bisphenol-A (BPA) levels in women undergoing infertility treatment and found a deleterious association between BPA and ovarian reserve (Souter *et al.,* 2013).

The main results reported by the above studies are further described throughout this review.

### Mechanisms involved in POI induced by endocrine disruptors

The first plausible mechanism to explain ovarian reserve disruption is the induction of increased apoptosis/atresia of primordial and primary follicles. Endocrine disruptors may induce oxidative stress and modifications in DNA methylation (Béranger *et al*., 2012; Vabre *et al*., 2017).

A second mechanism described as hypothetical associated premature ovarian insufficiency with increased follicular recruitment. Indirect impact derived from toxic agents may interfere local follicle stimulating hormone control, leading to rapid follicular stock reduction and increased risk of primary ovarian insufficiency (Béranger *et al*., 2012).

Toxicants may also cause the follicular pool to become refractory to maturation, thereby blocking follicular stimulation (Béranger *et al*., 2012).

The main toxicants and their respective likely modes of action are listed below (Table 1):

**Table 1:** summary of the main hormonal switches and their effects in the ovarian cycle

Chemical agents	Occupational exposure	Biological effect
2-bromopropane	Solvent in the electronic industry, intermediate agent in the chemical and pharmaceutical industries. Occupational exposure may occur through inhalation and dermal contact.	Toxic effect on follicular development.
Cadmium	80% of all cadmium is used to manufacture NiCd batteries. Other sources are zinc metallurgy, cadmium plating to protect metals from corrosion, pigments used in particular in plastics, ceramics, enamels, glass.	Statically significant correlation between elevated FSH and increased serum cadmium levels explained by the likely decrease in inhibin in more heavily exposed women.
7,12-Dimethylbenz[a]anthracene	Present in the environment through the combustion of organic material.	Degrades ovarian follicles in rats in a dose dependent manner, culminating with ovarian volume reduction.
4-Vinylcyclohexene	Chemical released in rubber tire, plasticizer, and pesticide manufacturing. Human exposure through skin contact, ingestion, and inhalation.	Degradation of the small preantral follicles (primordial and primary), leading to premature ovarian failure rats.
Triclosan	Used for over 40 years as an ingredient in products personal care, as detergents, soaps, lotions, toothpaste, and shampoos. Triclosan can also be used as an additive plastic, present in toys, medical devices, home, veterinary, and industrial products.	Increases levels of estrogen and modulates estrogen effects in target organs such as the uterus and ovaries. According to animal studies, it impairs blastocyst implantation in mice.
Methoxychlor	Former organochlorine insecticide (banned in Europe in 2002 and in the USA in 2004).	Exposure to this agent has caused a decrease in total ovary weight – a sign of follicular atresia.
Bisphenol A	Production of epoxy or polycarbonate resins.	Souter *et al*. (2013) reported a significant trend toward lower antral follicle counts in individuals with high urine levels of BPA.
Phthalates	Manufacturing of plastics in a wide spectrum of industrial applications. Human exposure to phthalates may occur through ingestion, inhalation, or skin contact.	Induces folliculogenesis disorders, which can lead to primordial follicle pool depletion by follicle recruitment acceleration.
Trichloroethylene	Used as an industrial solvent to clean metal parts, it is also present in many commercial products. Exposure may occur through inhalation of trichloroacetic acid, contaminated water intake, and transdermal absorption.	Laboratory administration in mice resulted in decreased oocyte fertilization and decreased plasma membrane protein binding to the oocyte.

#### 1) Increased apoptosis/atresia of primordial and primary follicles

1.a) 2-Bromopropane (2-BP): 2-BP is present in adhesive sprays and solvents; occupational exposure to 2-BP may occur either by inhalation or dermal contact (Béranger *et al*., 2012). Four studies have reported increased risk of POI from exposure to 2-BP. This agent has been associated with harmful effects on health - including impaired fertility - and linked to hematopoietic disorders and neurotoxicity (Béranger *et al*., 2012).

1.b) Cadmium: Cadmium is used predominantly in battery manufacturing. Other exposure sources are zinc metallurgy, some pigments (yellow-red) used in plastics, ceramics, enamel, glass materials, and materials resistant to high temperatures (Strauss *et al*., 2018). The National Survey of Health and Nutrition Evaluation Survey (NHANES III) found a statistically significant correlation between serum cadmium levels and increased FSH, explained by the probable decrease of inhibin in exposed women (Béranger *et al*., 2012).

1.c) 7,12-Dimethylbenz[a]anthracene (DMBA): DMBA is a member of the group of polycyclic aromatic hydrocarbons released into the environment by the combustion of organic material (Strauss *et al*., 2018). DMBA degrades ovarian follicles in rats in a dose-dependent manner, culminating with decreased ovarian volume (Béranger *et al*., 2012).

1.d) 4-Vinylcyclohexene (VCH): VCH is a chemical released during the manufacturing of rubber tires, plasticizers, and pesticides (Strauss *et al*., 2018). Human exposure to VCH occurs through dermal contact, ingestion, or inhalation (Strauss *et al*., 2018). VCH causes degradation of the small preantral follicles (primordial and primary), leading to premature ovarian failure in rats (Béranger *et al*., 2012).

1.e) Triclosan: this lipid soluble, broad spectrum antibacterial agent has been used for over 40 years as an ingredient in personal care products such as detergents, soaps, lotions, shampoos, and toothpaste (Strauss *et al*., 2018). Triclosan can also be used as a plastic additive in toys, medical devices, domestic, veterinary, and industrial products (Strauss *et al*., 2018). Triclosan increases estrogen levels and modulates the effect of estrogen on target organs such as the uterus and ovaries. According to animal studies, it impairs blastocyst implantation in mice (Béranger *et al*., 2012). An association between triclosan urine levels and treatment outcomes in women undergoing in vitro fertilization has been studied. Authors found that triclosan levels were associated with decreased oocyte yield, but not with other clinical IVF outcomes (implantation rate, pregnancy rate, or live birth rate) (Karwacka *et al.*, 2019).

1.f) Pesticides [Methoxychlor (MXC)]: Exposure to this organochlorine insecticide (banned in Europe in 2002 and in the USA in 2004) caused a decrease in total ovary weight in rats, in itself a sign of follicular atresia (Béranger *et al*., 2012).

#### 2) Inducers of increased follicle recruitment

2.a) Bisphenol-A (BPA): BPA was first synthesized in 1891 as a synthetic estrogen for the pharmaceutical industry, and has been used in the manufacturing of plastics (Krieg *et al.*, 2016). BPA was detected in various biological fluids, including urine, serum, saliva, follicular fluid, breast milk, umbilical cord blood, and amniotic fluid (Souter *et al.,* 2013). BPA is structurally similar to synthetic estrogen - diethylstilbestrol - and its estrogenic properties in vitro have been described in the literature. The results of in vitro and animal studies on the subject outlined possible risks to female reproductive health from exposure to BPA. These findings raised concerns that widespread and frequent exposure to BPA may adversely affect oocyte quality, follicular dynamics, ovarian reserve, and overall fertility (Souter *et al.,* 2013). Souter *et al.* (2013) evaluated the association of BPA with antral follicle count (AFC), day-3 serum follicle stimulating hormone levels (FSH), and ovarian volume in women undergoing infertility treatments. Higher urine BPA levels were associated with lower AFC, while no association was seen between BPA and FSH or ovarian volume. According to a review written by Karwacka *et al*. (2019), seven studies have examined the association between BPA and IVF outcomes. According to these studies, serum and urine BPA levels were correlated with decreased estradiol levels; fewer retrieved oocytes; and decreased oocyte maturity. On the other hand, no correlations were found between BPA and fertilization rate, proportion of high-quality embryos, implantation rate, clinical pregnancy rate, or live births. The EARTH trial found that BPA was associated with decreased antral follicle count measured by ultrasound on day 3 of the follicular phase of a woman’s unstimulated menstrual cycle (Ziv-Gal & Flaws, 2016; Messerlian *et al.*, 2018).

2.b) Phthalates: Phthalates are currently used in the manufacturing of plastics for a wide spectrum of industrial applications. Human exposure to phthalate may occur through ingestion, inhalation, or skin contact (Strauss *et al*., 2018). Phthalates exert a toxic effect on folliculogenesis, cause steroidogenesis disorders, and may lead to primordial follicle pool depletion by follicle recruitment acceleration (Béranger *et al*., 2012). According to the EARTH trial, higher urine levels of some phthalate metabolites were associated with reduced oocyte yield, lower likelihood of clinical pregnancy, increased risk of pregnancy loss, and lower likelihood of live birth following infertility treatment. (Hauser *et al.*, 2016; Messerlian *et al.*, 2016a; 2016b; 2018). The prospective study of Machtinger *et al.* (2018) found significant associations between urine phthalate metabolite levels and IVF outcomes. Specifically, higher urine concentrations of phthalate metabolites were negatively associated with total number of oocytes, mature oocytes, fertilized oocytes, and top-quality embryos. According to another prospective study, urine phthalate in women submitted to IVF was associated with increased biochemical pregnancy (RR 1.35; *p*=0.04), decreased clinical pregnancy rate (RR 1.56; *p*=0.006), and decreased live birth rate (RR 1.54; *p*=0.011) (Al-Saleh *et al.*, 2019).

#### 3) Follicular stimulation blockade

3.a) Polychlorinated biphenyls (PCB) [Trichloroethylene (TCE)]: TCE is a volatile chlorinated hydrocarbon that has detrimental effects on the ovary. TCE is used as an industrial solvent to clean metal parts. It is also present in many commercial products such as varnishes, finishes, lubricants, adhesives, paint removers, and cleaning products (Niederberger *et al.,* 2018). Laboratory administration of TCE to mice resulted in decreased oocyte fertilization and decreased protein binding to oocyte plasma membrane (Béranger *et al*., 2012). According to Bloom *et al.* (2017), women with higher polychlorinated biphenyl (PCB) levels in ovarian follicular fluid [151 (*p*=0.0003), 170 (*p*=0.02), and 180 (*p*=0.04)] had 47%, 32%, and 32% fewer baseline antral follicles, respectively. The authors also detected some associations between PCBs and clinical IVF outcomes. Higher PCB levels were associated with significantly lower implantation and live birth rates (Bloom *et al.*, 2017).

## CLOSING REMARKS

Environmental exposure to chemicals is clearly an important issue in the investigation of cases of POI (the cause of POI is unknown in 75% of the cases cause) (Béranger *et al*., 2012). A positive finding from this review is that most of the included studies included were well designed, based on prospective cohorts, and adjusted for potential confounders.

However, the included studies suffered from noteworthy limitations. The authors used a variety of biomarkers - urine, blood, and follicular fluids - to assess exposure, and these different biomarkers may have some bearing on the statistical associations described. Many studies used only one biological sample as a biomarker of exposure, which in case of chemicals with a short half-life may have resulted in substantial measurement error and attenuation of associations. Most of the presented studies had small sample sizes to observe the differences in effect estimated for the examined outcomes.

More studies are required to evaluate the effects of exposure on reproductive health, show dose-response effects, and assess the effects of longer-term exposure. For a better epidemiological and toxicological segmentation of future studies, it would be interesting to analyze the occupational exposure environments of patients with primary ovarian insufficiency. The goal would be to clarify the exposure to the agents mentioned in this review and, above all, systematically record suspected chemicals.

The impossibility of diagnosing POI at an earlier stage before irreversible damage to the follicular reserve sets in, makes the identification of potentially preventable risk factors even more urgent (Béranger *et al.,* 2012). In order to reinforce preventive activities, health professionals must be trained to detect patients exposed to harmful environments and at risk of early ovarian failure, in order to inform them about the risks and limit aggravating exposure.

**Figure 3:**
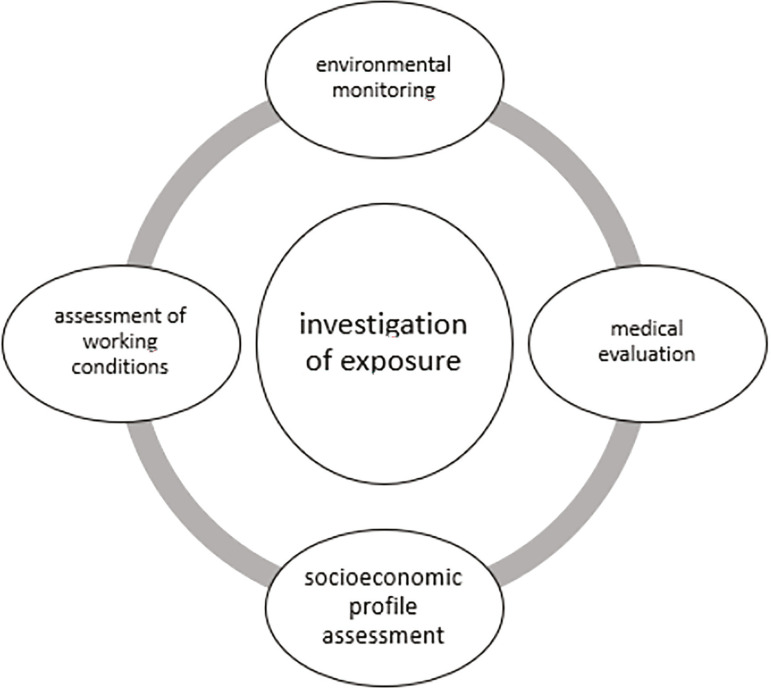
Integrated approach in an exposure assessment study (Adapted fromCarvalho *et al*. 2017)

The evaluation of occupational exposure by physicians in their private practices should include socioeconomic factors such as living conditions, housing, access to health services, and level of education of the patients (Figure 3) (Carvalho *et al.,* 2017). Patients at risk of exposure must be instructed by healthcare providers to wear personal protection equipment when handling toxic products, including the likes of aprons, rubber gloves, and goggles (Carvalho *et al.,* 2017). Endocrine disruptors pose serious threats to the health of humans and animals, with adverse effects extending into reproductive function. This is a growing public health concern that calls for protective and preventive measures, in addition to extensive communication with the population to mitigate exposure.

The data presented in this review showed the importance of organizing more prospective studies to shed light on the full extent of the impact of endocrine disruptors on ovarian and reproductive function.
